# Household Food Insecurity Risk and Weight Status Outcomes in Early Childhood: A Public Health Perspective

**DOI:** 10.3390/nu18121900

**Published:** 2026-06-12

**Authors:** Amanda Haboush-Deloye, Smriti Neupane, Gabriela Buccini

**Affiliations:** 1 Nevada Institute for Children’s Research and Policy, School of Public Health, University of Nevada Las Vegas, Las Vegas, NV 89119, USA; smriti.neupane@unlv.edu; 2 Department of Social and Behavioral Health, School of Public Health, University of Nevada Las Vegas, Las Vegas, NV 89119, USA; gabriela.buccini@unlv.edu

**Keywords:** household food insecurity, nutrition, underweight, obesity, body mass index, kindergarten, children

## Abstract

Background: Household food insecurity (HFI), defined as the lack of reliable access to adequate food because of limited money or resources, may influence children’s nutritional status. This study aimed to examine the association between HFI risk, based on a single screening item, and underweight and obesity among kindergarten children in Nevada. Methods: Cross-sectional data from the Kindergarten Health Survey (KHS) collected across three school years (2022–2023, 2023–2024, and 2024–2025) were analyzed using a pooled sample of 7267 children. HFI risk was assessed using one item from the Hunger Vital Sign. Weight status was determined using Body Mass Index (BMI) guidelines from the Centers for Disease Control and Prevention (CDC). Descriptive statistics and multinomial logistic regression examined associations between HFI risk and underweight and obesity, adjusting for confounders. Results: Across the pooled sample, 16.3% were at risk for HFI, 16.0% were underweight, and 21.9% had obesity. In pooled analysis, HFI risk was associated with higher odds of obesity (Adjusted Odds Ratio [AOR] 1.29; 95% Confidence Interval [CI]: 1.05–1.59), but not underweight, compared with food-secure children. In year-specific analyses, higher odds of underweight were observed in 2023–2024 (AOR 1.74; 95% CI: 1.14–2.66) and 2024–2025 (AOR 1.58; 95% CI: 1.04–2.38). Conclusions: HFI risk was associated with obesity among kindergarten children in Nevada, while associations with underweight were observed only in certain school years and should be interpreted cautiously. These findings suggest HFI risk as an important early childhood health concern and support the need for nutrition support, family assistance, and longitudinal research.

## 1. Introduction

Body Mass Index (BMI) is a measure of a person’s weight relative to their height [1]. It is commonly used to assess whether a child’s weight is appropriate for their age and height. Based on BMI, children can be classified as underweight, healthy weight, overweight, or obesity [1]. In the United States of America (USA), childhood obesity is a growing public health concern, beginning as early as preschool years and continuing through school age. Currently, obesity affects 13.4% of children aged 2 to 5 years, and nearly one in five children and adolescents aged 2 to 19 has obesity [2]. These conditions increase the risk of type 2 diabetes, asthma, sleep disturbances, joint issues, gallstones, anxiety, depression, low self-esteem, bullying, and obesity later in adulthood [3]. In contrast, 3.04% of children aged 2 to 5 years and 4.1% of children and adolescents aged 2 to 19 years in the USA are underweight [4]. Although less common, undernutrition in children can still signal malnutrition, which can lead to lowered immunity, delayed motor skills, and reduced school readiness. In Nevada specifically, 22.2% of kindergarten children have obesity, and 16.2% are underweight [5].

Household food insecurity (HFI) refers to the condition of not having reliable access to adequate food due to financial or resource constraints [6]. In the USA, 47.4 million people live in food-insecure households, including 17.9% of households with children, approximately 6.5 million families [6]. In Nevada, 136,580 children (20%) face food insecurity [7]. Food insecurity may contribute to malnutrition, which is not only about insufficient food but also limited access to nutritious food. For kindergarten-aged children, who are in a critical developmental stage, food insecurity can cause developmental delays, anemia, poor academic performance, behavioral issues, anxiety, depression, oral health problems, and impaired growth [8].

Food insecurity has a complex relationship with children’s weight status [9,10,11,12]. It can influence eating behaviors in ways that contribute to both underweight and obesity. On one hand, food insecurity is associated with undernutrition, as children in food-insecure households may not consume enough calories to meet their daily energy needs, which can hinder growth and lead to underweight [11]. On the other hand, families experiencing food insecurity often rely on cheaper, calorie-dense but nutrient-poor foods, such as sugary beverages and processed snacks, and may have limited access to fresh fruits and vegetables. This leads to poor diet quality and increases the risk of obesity [9,12]. This phenomenon suggests the dual burden of malnutrition, where food insecurity may result in both underweight and obesity, depending on the severity and types of food available [10]. Some studies have reported higher odds of underweight among children in food-insecure households [13,14], while others have found higher odds of obesity [15]. While the link between food insecurity and childhood obesity is fairly well-established, the relationship with underweight remains less consistent, particularly in high-income countries.

There is limited research exploring the association between HFI and both underweight and obesity among kindergarten-aged children in the USA [16]. Early childhood is a critical period to understand how nutritional and environmental factors interact and affect long-term health outcomes. Examining this relationship can guide public health leaders and educators in developing strategies to improve child nutrition and overall well-being [17]. In this study, we hypothesized that risk for HFI increases the risk of both underweight and obesity.

Using cross-sectional data from three school years (2022–2023, 2023–2024, and 2024–2025), representing a statewide sample of children entering kindergarten in Nevada, this study aimed to examine the association between risk for HFI and weight status. While most previous studies have focused primarily on obesity, often overlooking underweight [11], our study contributes to addressing this gap by examining both forms of malnutrition. By focusing on this vulnerable age group, this study provides additional evidence on the broader implications of the risk of HFI on child health and offers relevant insights to inform policies and interventions aimed at promoting healthy development during this formative stage.

## 2. Materials and Methods

### 2.1. Study Design and Setting

The Kindergarten Health Survey (KHS) is an annual cross-sectional survey designed to assess the overall health status of children entering kindergarten across the state, done in collaboration between the Clark County School District and the Southern Nevada Health District in Nevada, USA. The KHS is a self-reported survey consisting of 37 questions, 12 addressing socio-demographic information and 25 related to the child’s health. Parents complete the survey on behalf of their children.

### 2.2. Data Collection

Data collection occurs each school year from August through October, at the beginning of the fall semester. The KHS is distributed to kindergarten teachers in nearly all public elementary schools across Nevada’s 17 school districts, with additional participation from some private and charter schools. The survey was available only in paper format. Parents received a single-page, double-sided questionnaire with English on one side and Spanish on the other, reflecting the two most commonly spoken languages in Nevada [18]. Completed surveys were returned either to the child’s teacher, the school office, or mailed directly to the Nevada Institute of Children’s Research and Policy (NICRP), the coordinating research institution. Online submissions were not available.

Parents who chose to participate answered questions about socio-demographics, health insurance, food insecurity, health care access, barriers to care, breastfeeding status, and various child health conditions and behaviors. For this study, data from three school years (2022–2023, 2023–2024, and 2024–2025) were included in the analysis.

### 2.3. Sample

Eligible responses included parents who completed the KHS for children aged 4–6 years entering kindergarten. Children younger than four or older than six, who were unlikely to be in kindergarten, were excluded from the study.

According to the Nevada Department of Education, 31,951 children were enrolled in kindergarten during the 2022–2023 school year, 28,931 during the 2023–2024 school year, and 30,947 during the 2024–2025 school year [19]. A total of 16,583 parents completed the KHS during three school years. Of these, 9316 responses (56.2%) were excluded from the analysis because of invalid or incomplete BMI (height/weight) data. To improve data accuracy and avoid overestimating weight-status outcomes, strict guidelines were applied when reviewing BMI data. Responses were excluded for a few reasons: First, if the parent reported that the child was younger than 4 or older than 6 years, since age is crucial for determining BMI and weight status. Second, if the child’s reported height fell outside of the 95th percentile for children aged 4–6 years, based on CDC reference standards [1]. Third, if the child’s reported weight was under 20 pounds, as it would not yield a valid BMI. The final pooled analytical sample included 7267 valid responses: 2510 from school year 2022–2023; 2347 from school year 2023–2024; and 2410 from school year 2024–2025.

A sensitivity analysis comparing participants included in the analytic sample with those excluded due to invalid or incomplete BMI data found statistically significant differences in several characteristics, including risk for HFI based on a single screening item (16.4% vs. 25.7%, *p* < 0.001), housing status (renters: 41.8% vs. 55.5%, *p* < 0.001), single-parent households (22.4% vs. 31.5%, *p* < 0.001), household income (<$34,999: 16.0% vs. 30.5%, *p* < 0.001), and child race (non-White: 55.9% vs. 73.5%, *p* < 0.001) (Supplementary Table S4). While these differences may limit the generalizability of the findings, the analytic sample still remains sufficiently robust to provide meaningful insights into the relationship between children’s weight status and risk for HFI based on a single screening item.

### 2.4. Measurements

Outcome: Children’s weight status was the primary outcome of interest. Data were collected from parent-reported responses on their child’s height (in feet and inches, converted into inches) and weight (in pounds). BMI was calculated for each child with valid height and weight data using the Centers for Disease Control and Prevention (CDC) recommended formula:

BMI = [(Weight in pounds)/(Height in inches)^2^] × 703

Based on BMI, along with the child’s age and gender, weight status categories were assigned according to CDC standards: underweight, healthy weight, overweight, and obesity [1].

Independent Variable: Risk for HFI was the independent variable, measured using one item from the ‘Hunger Vital Sign’, which is based on the U.S. Household Food Security Survey Module (HFSSM) [20]. Although the ‘Hunger Vital Sign’ includes two items, this study only used one item as a proxy to assess risk for HFI. The KHS included only this single question because of space limitations, as the survey was restricted to one page. This decision was made to reduce the burden on parents and help improve response rates. In addition, previous studies have also used a single-item food insecurity question to assess the risk of food insecurity [21,22,23,24,25]. The survey question was: “Within the past 12 months, the food we bought just didn’t last, and we didn’t have money to get more.” Response options were “Often true,” “Sometimes true,” and “Never true.” Parents who answered “Often true” or “Sometimes true” were categorized as at risk for HFI based on a single screening item.

Covariables: Covariables were grouped into different categories based on their influence on the outcome. Household characteristics included housing status (rent; own), single parent (yes; no), household income (less than $34,999; $35,000–$74,999; more than $75,000), child race (White; others), and child insurance (insured; uninsured). The “others” race category included African American, Asian, Hispanic/Latino, Native American/Alaska Native, Pacific Islander, multiple races, and other racial groups not listed above. Healthcare utilization included whether the child had a primary care provider (yes; no) and whether the child had gone for a routine check-up during the past 12 months (yes; no). Social determinants of health captured whether parents experienced at least one barrier to accessing healthcare for their child (yes; no). Child well-being included physical activity (meeting recommendations) (yes; no). Children were considered to meet recommendations if they engaged in at least 60 min of physical activity per day [26]. Breastfeeding status included exclusive breastfeeding at 6 months (yes; no), and continued breastfeeding at 12 months (yes; no). 

### 2.5. Statistical Analysis

Analyses were conducted using Statistical Package for the Social Sciences (SPSS), Version 29. Statistical analysis was performed for each survey wave and for the pooled sample. Descriptive analyses were conducted to summarize the frequencies and percentages of the outcome, independent variable, and covariables. Bivariate analyses were performed using chi-square tests to examine associations between the independent variable and covariables with the outcome, and also to identify variables for inclusion in the multinomial logistic regression model. Variables with a *p*-value < 0.20 in the bivariate analyses were included in the multinomial logistic regression model.

Multinomial logistic regression analyses were conducted to examine the association between HFI risk based on a single screening item and weight status categories (underweight, healthy weight, overweight, and obesity), while adjusting for potential confounders. Adjusted odds ratios (AORs) and their corresponding 95% confidence intervals (CIs) were estimated to assess the association between risk for HFI based on a single screening item and child weight status. A *p*-value of <0.05 was used as the criterion for statistical significance.

## 3. Results

Table 1 presents descriptive characteristics for the pooled sample and for each survey wave, including the outcome, independent variable, and covariables. Among all children (pooled sample), 16.0% were underweight, 51.7% had a healthy weight, 10.4% were overweight, and 21.9% had obesity. Overall, approximately one out of every six children (16.3%) was at risk for HFI based on a single screening item. Most children were non-white (55.9%), less than half (43.4%) met the recommended level of physical activity for children under five, 30.7% were exclusively breastfeeding at 6 months, and 39.9% continued breastfeeding at 12 months (Table 1).

Bivariate analysis for the pooled sample showed that obesity was more common among children in food-insecure households (30.0%) than in food-secure households (20.3%). Those who lived in rented homes, had a single parent, belonged to non-white racial groups, faced barriers to healthcare, did not meet physical activity recommendations, were not exclusively breastfed at 6 months, and did not continue breastfeeding at 12 months were more likely to have obesity than those who lived in their own homes, had married parents, were white, faced no barriers to healthcare, met physical activity recommendations, were exclusively breastfed at 6 months, and continued breastfeeding at 12 months, respectively (Table 2). At the same time, there was no significant difference in the prevalence of underweight between children from food-secure and food insecure households. Detailed bivariate analysis results for individual school years, 2022–2023, 2023–2024, and 2024–2025 are presented in Supplementary Tables S1, S2, and S3, respectively. 

**Figure 1:**
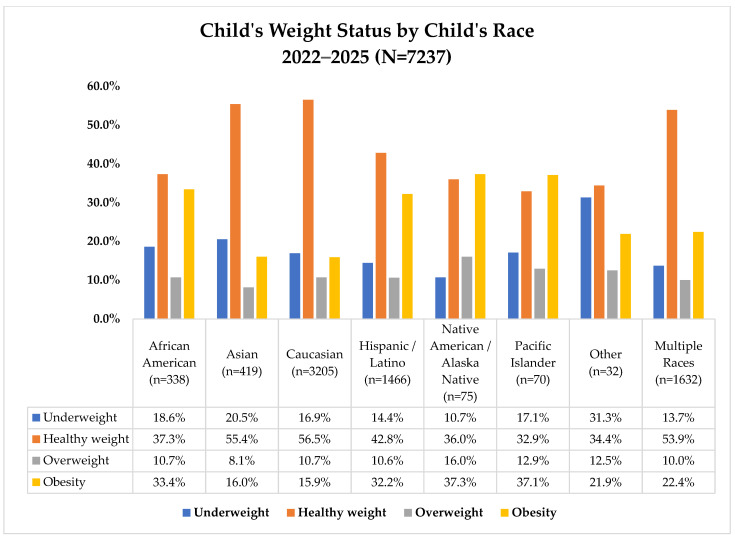
Child’s weight status by child’s race, 2022–2025 (*N* = 7237).

Figure 1 illustrates children’s weight status by racial/ethnic group in the pooled sample. In the “Other” race category, underweight was the most common weight status (31.3%), while most Caucasian children (56.5%) and Asian children (55.4%) were in the healthy weight category. Obesity was highest among Native American/Alaska Native children (37.3%) and Pacific Islander children (37.1%), followed by African American children (33.4%) and Hispanic/Latino children (32.2%).

Pooled multinomial logistic regression analysis showed no association between risk for HFI based on a single screening item and underweight when all three school years were analyzed together. However, risk for HFI based on a single screening item was associated with obesity, with children experiencing risk for HFI based on a single screening item having 29% higher odds of obesity compared to food-secure children (AOR 1.29; 95% CI: 1.05–1.59). When examined by individual school year, risk for HFI based on a single screening item was associated with higher odds of underweight. Specifically, the odds of being underweight were 74% higher in the 2023–2024 school year (AOR 1.74; 95% CI: 1.14–2.66) and 58% higher in the 2024–2025 school year (AOR 1.58; 95% CI: 1.04–2.38) compared to food-secure children (Table 3).

## 4. Discussion

Our study examined the relationship between risk for HFI based on a single screening item and children’s weight status among kindergarten children in Nevada, USA. We found that the risk for HFI based on a single screening item was linked to higher odds of obesity but not underweight in the pooled analysis. Only associations with underweight were observed in some year-specific analyses. Using three years of statewide child health survey data, this study makes an important contribution by suggesting how limited food access may affect children’s nutrition, growth, and development. To our knowledge, this is one of the first studies to examine the coexistence of underweight and obesity among young children in Nevada, USA, in relation to HFI risk.

In our study, the pooled sample showed that the prevalence of underweight among kindergarten children in Nevada (16.0%) was substantially higher than the national prevalence of 3.4% for children aged 2–5 years and 3.6% for children aged 6–11 years [4]. These findings should be interpreted cautiously because height and weight data were parent-reported rather than directly measured, which may have introduced reporting errors and misclassification, particularly among young children. However, these data are still important, especially given the limited number of studies focused on this age group. Many existing studies use direct height and weight measurements, but primarily focus on overweight and obesity [27,28,29]. Underweight is often either not reported separately or grouped with normal weight as BMI below the 85th percentile. Therefore, while these findings may partly reflect limitations in the parent-reported anthropometric data, they still provide important public health information and support the need for future studies using directly measured height and weight with validated assessment methods.

On the other hand, the prevalence of obesity in our pooled sample (21.9%) was higher than the national prevalence of 14.4% for children aged 2–4 years [30]. It was similar to national estimates among older children, where obesity affects 20.3% of children aged 6–11 years and 21.2% of adolescents aged 12–19 years [2]. Previous Nevada reports showed that the prevalence of obesity among kindergarten children was 18.7% in 2021 and 22.2% in 2022 [5], which is consistent with our findings. Childhood obesity is also a global public health concern, although rates vary widely across countries and regions. According to the World Health Organization (WHO), the global prevalence of obesity among children and adolescents aged 5–19 years was 8.2%, with higher levels reported in countries such as the United States (20.6%), Australia (15.8%), Brazil (15.5%), and China (11.9%), and lower levels in India (3.4%), Ethiopia (1.7%), France (4.1%), and Japan (4.4%) [31]. For children under 5 years of age, the United Nations Children’s Fund (UNICEF) reports a lower global obesity prevalence of about 1% [32]. These patterns suggest that obesity becomes more common as children grow older, while also showing that the burden varies across settings. Similarly, in Indonesia, 10.8% of children under five were underweight, and only 4.2% had obesity [33], suggesting that Nevada’s rates of both undernutrition and overnutrition are relatively high in comparison. 

The present study reported that almost one in six kindergarten children lived in households at risk for food insecurity based on a single screening item, a prevalence that is similar to the national rate (17.9%) in the United States [6], but slightly lower than a study among preschool children, which reported 20% experiencing household food insecurity [34]. Another study found even higher rates, with 27.3% of households experiencing food insecurity [35]. Surprisingly, an additional study in the U.S. found that nearly three-quarters of families were food insecure, which was over four times higher than our findings [15]. The higher rate in that study was particularly due to the focus on Hispanic families, who often face greater economic challenges, limited access to affordable, healthy foods, and structural barriers that exacerbate food insecurity [15]. As Nevada is one of the most diverse states in the country, these findings suggest the importance of culturally relevant efforts to improve food access and food security among minority populations.

Consistent with previous studies and reports, our study also found differences in children’s weight status across racial and ethnic groups [5,15,36,37]. Minority groups, including Native Americans, Pacific Islanders, African Americans, and Hispanics, were more likely to have obesity compared to White/Caucasian children. These disparities may be linked to factors such as lower household income, low education among parents, and the added stress associated with racial discrimination, all of which influence how parents practice responsive feeding, encourage physical activity, and find the time or resources to make healthy decisions about their children’s diet and well-being [38].

While several studies did not find an association between food insecurity and underweight [35,39,40], our pooled analysis also did not show a significant association between HFI risk based on a single screening item and underweight. However, year-specific analyses showed that children living in food-insecure households based on a single screening item had higher odds of being underweight during the 2023–2024 and 2024–2025 school years. This aligns with other studies that reported a similar relationship [13,33], and a recent systematic review also confirmed that HFI is significantly associated with underweight and stunting in children [14]. These year-specific findings should be interpreted cautiously. Most of the existing studies on this topic have been conducted in low-income countries, where undernutrition is more common. Still, our findings provide an important reminder that risk of HFI may also contribute to underweight, even in high-income countries such as the United States, as classified by the World Bank based on national income indicators [41]. Although the national prevalence of underweight is relatively low, it should not be overlooked by local leaders and policymakers, as even a small proportion of affected children face serious risks to healthy growth and development.

Another key finding was that children at risk for HFI based on a single screening item were more likely to have obesity in the pooled sample when all three school years were considered together. This result is consistent with several other studies that have reported a similar link between food insecurity and higher odds of obesity [15,33,42]. One possible explanation is that food-insecure households often have limited access to healthy foods, leading to diets of lower quality and less variety. Food-insecure households often rely on home-cooked meals made with inexpensive, calorie-dense ingredients such as refined grains and processed foods, which may lead to metabolic changes and affect weight gain over time. Parents in food-insecure households may also allow children to eat more when food is available to protect them from future starvation, leading to excessive calorie intake. At the same time, children in these households may spend more time in sedentary activities, such as television or phone use, which further contributes to weight gain. These findings support the need for interventions that both improve access to nutritious foods and promote physical activity to address the dual challenges of food insecurity and childhood obesity [42].

However, not all studies have found an association between food insecurity and childhood obesity. Several studies reported no clear relationship [12,34,35]. Interestingly, one of the studies did find a link among older children (6–11 years), but not among preschool-aged children (2–5 years) [12]. One possible reason is that preschool-aged children may benefit from programs like the Child and Adult Care Food Program (CACFP), which provides balanced meals regardless of household food insecurity, helping buffer its impact on weight status [34]. Another explanation for these inconsistent findings may be differences in how food insecurity was measured across studies. Our study, for example, used a single-item measure from the Hunger Vital Sign, while others relied on more comprehensive tools. Taken together, these findings suggest that the relationship between HFI risk and children’s weight status may vary across different weight outcomes. Further longitudinal research may help better understand these associations over time.

### Limitations

This study has several limitations that should be considered when interpreting the findings. First, more than half of the original responses were excluded because of invalid or incomplete BMI-related data. Sensitivity analyses indicated that excluded participants were more likely to experience food insecurity based on a single screening item, have lower household incomes, identify as racial or ethnic minorities, and live in single-parent households, suggesting that missingness was not completely random. Differential missingness in parent-reported height and weight data has been documented in previous population-based research and may reflect differences in knowledge of current height or weight, comfort disclosing anthropometric information, survey completion patterns, or other socioeconomic factors [43,44]. As a result, selection bias is possible, and the observed associations may not fully represent those among excluded participants. If the relationship between HFI risk and child weight status differs among families with missing anthropometric data, the magnitude of the observed associations may have been either underestimated or overestimated. However, the final analytic sample remained large and demographically diverse, providing substantial statistical power and representation across key population groups.

Second, the survey was voluntary, which may have introduced participation bias. Families who were more organized, had higher educational attainment, were more engaged in their child’s education, or felt more comfortable completing surveys may have been more likely to participate. In addition, HFI was self-reported and may have been underreported because some families may feel uncomfortable answering questions about food access or household needs. Stigma, cultural norms, or fear of judgment may affect how families respond to these questions. Future surveys should use clear, culturally responsive wording so that families feel safe and comfortable providing accurate information. Third, child height and weight data were parent-reported and therefore subject to recall error, reporting bias, and potential misclassification of BMI categories. Furthermore, BMI was used as the primary indicator of nutritional status and does not distinguish between fat mass and lean body mass.

Fourth, HFI risk was assessed using a single item from the Hunger Vital Sign rather than the validated two-item screening tool, which may not have fully captured the multidimensional nature of HFI. Fifth, race-specific findings should be interpreted cautiously because some racial and ethnic groups had comparatively smaller sample sizes, which may have resulted in less stable estimates.

Sixth, most participating schools were public schools, while private and charter schools were underrepresented, and the sample was limited to kindergarten children in Nevada, which may limit generalizability to other populations. Additionally, because data were collected from multiple schools and districts, responses may not have been fully independent. As clustering was not accounted for in the analyses, standard errors may have been underestimated.

Finally, the cross-sectional design precludes conclusions regarding causality or the temporal ordering of associations between HFI risk and child weight status. Some inconsistencies between pooled and school-year-specific analyses also suggest the possibility of temporal or contextual variation in these relationships. Despite these limitations, the study provides important public health information regarding the relationship between HFI risk and child weight status among kindergarten children in Nevada.

Taken together, these findings suggest possible coexisting risks of underweight and obesity among young children in Nevada experiencing HFI. This indicates that HFI risk may be associated with multiple forms of nutritional risk among children. These challenges carry important public health implications, as children who face these challenges early in life may have lasting implications for health and development. Tackling HFI requires more than increased food access; it also calls for better quality and diversity of foods, culturally tailored interventions, nutrition education, and family-based approaches. Schools and community programs also play a key role in providing balanced meals and encouraging physical activity. Given Nevada’s racial and ethnic diversity, strategies that are responsive to cultural and socioeconomic contexts are especially relevant. Because this study is cross-sectional, the findings cannot establish causality. Further longitudinal research is needed to better understand the pathways linking HFI with weight outcomes and to inform effective and sustainable interventions.

Our findings also have important implications for clinical practice and public health policy. First, pediatric providers should routinely screen for HFI. When food insecurity is identified, providers should connect families with resources such as school meal programs, nutrition assistance programs, and local food banks. Second, public health efforts should focus on ensuring that food-insecure families have access to nutritious and affordable foods, such as through incentives for buying fruits and vegetables and providing nutrition education on healthy, budget-friendly meals. Third, policies should strengthen child nutrition programs, and early childhood programs should be required to provide balanced and nutritious meals for all children, which may help protect children’s nutritional outcomes.

## 5. Conclusions

Risk for HFI based on a single screening item was associated with obesity among kindergarten children in Nevada, USA, in the pooled analysis. Associations with underweight were observed only in specific school years and should be interpreted cautiously. Overall, these findings suggest a potential relationship between HFI and children’s nutritional status; however, the results should be considered in light of the study limitations. Improved access to U.S.-based school meal programs, such as the National School Lunch Program and School Breakfast Program, along with the Supplemental Nutrition Assistance Program, local school wellness policies, and early childhood nutrition support strategies, may help improve food access, support healthy growth, and reduce nutrition-related health disparities among children.

### Future Perspectives

Future studies should use longitudinal designs along with validated anthropometric and food insecurity measures to better understand how HFI influences child growth over time. Examining factors such as dietary patterns, food quality, physical activity, household food environments, and socioeconomic conditions may help explain why food insecurity is linked with both obesity and underweight. Further analyses by sex, race/ethnicity, and socioeconomic status may also help identify populations at greater risk for health disparities. In addition, studies conducted in different geographic and cultural settings may improve the generalizability of findings and help inform more targeted nutrition and public health interventions.

## Figures and Tables

**Table 1:** Descriptive characteristics of children by survey wave, 2022–2025.

	2022–2025 (*n* = 7267)	2022–2023 (*n* = 2510)		2023–2024 (*n* = 2347)		2024–2025 (*n* = 2410)	
	%	No.	%	No.	%	No.	%
Outcome							
Children’s Weight Status							
Underweight	16.0	405	16.1	392	16.7	365	15.1
Healthy weight	51.7	1275	50.8	1207	51.4	1272	52.8
Overweight	10.4	276	11.0	240	10.2	243	10.1
Obesity	21.9	554	22.1	508	21.6	530	22.0
Independent Variable							
Household Food Insecurity (HFI) Risk							
Food Insecure	16.3	390	15.7	387	16.7	399	16.7
Food Secure	83.7	2094	84.3	1936	83.3	1989	83.3
Household Characteristics							
Housing Status							
Rent	41.8	1026	42.3	933	41.0	986	42.2
Own	58.2	1402	57.7	1342	59.0	1352	57.8
Single Parent							
Yes	22.4	543	22.5	498	22.2	523	22.6
No	77.6	1875	77.5	1742	77.8	1792	77.4
Household Income							
Less than $34,999	16.0	352	16.6	327	16.3	308	15.0
$35,000–$74,999	28.9	646	30.4	582	29.0	558	27.2
More than $75,000	55.2	1125	53.0	1096	54.7	1189	57.9
Child Race							
White	44.1	1174	46.8	1032	44.0	999	41.5
Non-White	55.9	1336	53.2	1315	56.0	1411	58.5
Child Insurance							
Insured	95.8	2406	96.2	2222	95.2	2300	96.1
Uninsured	4.2	96	3.8	113	4.8	94	3.9
Healthcare Utilization							
Primary Care Provider							
Yes	93.1	2333	94.1	2119	91.4	2234	93.6
No	6.9	146	5.9	200	8.6	153	6.4
Routine Check Up							
Yes	92.2	2293	92.4	2106	90.9	2218	93.3
No	7.8	188	7.6	211	9.1	160	6.7
Social Determinants of Health							
Any Barriers Accessing Health Care							
Yes	18.9	474	19.2	430	18.6	447	18.9
No	81.1	1995	80.8	1877	81.4	1922	81.1
Child Well-being							
Physical Activity (Meeting Recommendations)							
Yes	43.4	1046	42.7	1027	44.8	1011	42.9
No	56.6	1404	57.3	1267	55.2	1345	57.1
Breastfeeding Status							
Exclusive Breastfeeding (6 months)							
Yes	30.7	748	31.7	652	29.7	687	30.4
No	69.3	1610	68.3	1542	70.3	1570	69.6
Continued Breastfeeding at 12 months							
Yes	39.9	935	38.8	925	41.0	922	40.0
No	60.1	1475	61.2	1330	59.0	1382	60.0

**Table 2:** Bivariate analysis of weight status of children in the pooled sample, 2022–2025 (*N* = 7267).

	Underweight	Healthy Weight	Overweight	Obesity	*p*-Value
	%	%	%	%	
Household Food Insecurity (HFI) Risk (*n* = 7195)					
Food Insecure	16.9	42.3	10.8	30.0	<0.001 *
Food Secure	15.9	53.5	10.3	20.3	
Housing Status (*n* = 7041)					
Rent	15.8	45.1	10.7	28.4	<0.001 *
Own	16.0	56.6	10.4	17.0	
Single Parent (*n* = 6973)					
Yes	15.8	41.5	12.2	30.5	<0.001 *
No	16.2	54.7	9.9	19.2	
Household Income (*n* = 6183)					
Less than $34,999	14.9	41.7	10.0	33.3	<0.001 *
$35,000–$74,999	16.5	47.9	10.6	25.0	
More than $75,000	15.6	57.3	10.3	16.8	
Child Race (*n* = 7267)					
White	16.9	56.5	10.7	15.9	<0.001 *
Non-White	15.3	47.9	10.2	26.7	
Child Insurance (*n* = 7231)					
Insured	16.1	51.9	10.5	21.5	0.045 *
Uninsured	14.9	47.5	9.2	28.4	
Primary Care Provider (*n* = 7185)					
Yes	16.0	52.2	10.4	21.4	0.002 *
No	15.8	45.1	10.6	28.5	
Routine Check Up (*n* = 7176)					
Yes	15.8	52.3	10.5	21.4	0.010 *
No	18.1	45.6	10.2	26.1	
Any Barriers Accessing Health Care (*n* = 7145)					
Yes	14.7	50.1	9.8	25.5	0.004 *
No	16.3	52.1	10.6	21.0	
Physical Activity (Meeting Recommendations) (*n* = 7100)					
Yes	15.5	54.8	10.7	19.0	<0.001 *
No	16.3	49.4	10.0	24.2	
Exclusive Breastfeeding at 6 months (*n* = 6809)					
Yes	16.7	56.4	10.1	16.8	<0.001 *
No	15.8	50.0	10.7	23.6	
Continued Breastfeeding at 12 months (*n* = 6969)					
Yes	16.6	56.0	9.5	18.0	<0.001 *
No	15.5	49.7	11.1	23.8	

* *p* < 0.05.

**Table 3:** Multinomial logistic regression analysis on the relationship between risk for household food insecurity (HFI) based on a single screening item and weight status of children, 2022–2025.

	Pooled Sample 2022–2025 (AOR, 95% CI) ^a^			2022–2023 (AOR, 95% CI) ^b^			2023–2024 (AOR, 95% CI) ^c^			2024–2025 (AOR, 95% CI) ^d^		
	UW	OW	OB	UW	OW	OB	UW	OW	OB	UW	OW	OB
Food Secure	Ref	Ref	Ref	Ref	Ref	Ref	Ref	Ref	Ref	Ref	Ref	Ref
Food Insecure	1.23 (0.96, 1.57)	1.08 (0.81, 1.46)	1.29 * (1.05, 1.59)	0.59 (0.38, 0.94)	0.98 (0.61, 1.57)	1.01 (0.71, 1.42)	1.74 * (1.14, 2.66)	0.89 (0.50, 1.59)	1.37 (0.94, 1.99)	1.58 * (1.04, 2.38)	1.44 (0.89, 2.34)	1.41 (0.99, 2.02)

Abbreviations: UW, Underweight; OW, Overweight; OB, Obesity; CI, Confidence Interval; Ref, Reference category; ^a^ Model adjusted for housing status, single parent, household income, child race, child insurance, primary care provider, routine checkup, any barriers accessing health care, physical activity, exclusive breastfeeding at 6 months, breastfeeding at 12 months; ^b^ Model adjusted for housing status, single parent, household income, child race, child insurance, primary care provider, physical activity, exclusive breastfeeding at 6 months, breastfeeding at 12 months; ^c^ Model adjusted for housing status, single parent, household income, child race, child insurance, any barriers accessing health care, physical activity, exclusive breastfeeding at 6 months, breastfeeding at 12 months; ^d^ Model adjusted for housing status, single parent, household income, child race, primary care provider, routine checkup, any barriers accessing health care, physical activity, exclusive breastfeeding at 6 months, breastfeeding at 12 months. * *p* < 0.05.

## Data Availability

The data supporting this study are available within the manuscript.

## Supplementary Materials

The following supporting information can be downloaded at: https://www.mdpi.com/article/10.3390/nu18121900/s1, Table S1: Bivariate analysis of weight status of children in the school year 2022–2023 sample (*N* = 2510); Table S2: Bivariate analysis of weight status of children in the school year 2023–2024 sample (*N* = 2347); Table S3: Bivariate analysis of weight status of children in the school year (2024–2025) sample (*N* = 2410); Table S4: Comparison of sociodemographic characteristics between excluded and included participants in the pooled sample (2022–2025).

## Abbreviations

The following abbreviations are used in this manuscript:

HFI	Household Food Insecurity
BMI	Body Mass Index
CDC	Centers for Disease Control and Prevention
AOR	Adjusted Odds Ratio
CI	Confidence Interval
USA	United States of America
NICRP	Nevada Institute for Children’s Research and Policy
KHS	Kindergarten Health Survey
HFSSM	Household Food Security Survey Module
SPSS	Statistical Package for the Social Sciences
UW	Underweight
OW	Overweight
OB	Obesity
UNICEF	United Nations Children’s Fund
WHO	World Health Organization
