# Supplementary material for: Household Food Insecurity Risk and Weight Status Outcomes in Early Childhood: A Public Health Perspective
Source: Nutrients. 2026 Jun 12;18(12):1900. doi: 10.3390/nu18121900 (PMC13306024; doi:10.3390/nu18121900)
Supplement: Supplementary file 1 [file nutrients-18-01900-s001.zip › nutrients-4320164-supplementary.pdf]

Supplementary Materials

Table S1. Bivariate analysis of weight status of children in the school year 2022–2023 sample (*N*=2510).

	Underweight %	Healthy weight %	Overweight %	Obesity %	p-value
Household Food Insecurity (HFI) Risk (*n*=2484)					
Food Insecure	14.4	45.4	10.8	29.5	0.002*
Food Secure	16.6	51.8	10.8	20.8	
Housing Status (*n*=2428)					
Rent	16.3	43.0	11.2	29.5	<0.001*
Own	15.5	56.8	11.1	16.6	
Single Parent (*n*=2418)					
Yes	15.8	40.1	13.1	30.9	<0.001*
No	16.1	54.2	10.2	19.5	
Household Income (*n*=2123)					
Less than $34,999	14.2	42.0	10.2	33.5	<0.001*
$35,000-$74,999	17.8	47.2	12.4	22.6	
More than $75,000	15.5	55.8	10.7	18.0	
Child Race (*n*=2510)					
White	16.6	55.0	11.8	16.6	<0.001*
Non-White	15.7	47.1	10.3	26.9	
Child Insurance (*n*=2502)					
Insured	16.2	51.2	11.1	21.6	0.05*
Uninsured	14.6	42.7	9.4	33.3	
Primary Care Provider (*n*=2479)					
Yes	16.2	51.2	11.1	21.5	0.045*
No	13.7	45.2	9.6	31.5	
Routine Check Up (*n*=2481)					
Yes	16.3	50.8	11.2	21.8	0.628
No	14.4	52.1	9.0	24.5	
Any Barriers Accessing Health Care (*n*=2469)					
Yes	16.0	48.7	10.5	24.7	0.498
No	16.3	51.2	11.0	21.5	
Physical Activity (Meeting Recommendations) (*n*=2450)					
Yes	15.6	53.0	12.0	19.4	0.014*
No	16.6	49.2	10.0	24.2	
Exclusive Breastfeeding at 6 months (*n*=2358)					
Yes	15.1	58.3	10.6	16.0	<0.001*
No	16.8	47.8	11.1	24.3	
Breastfeeding at 12 months (*n*=2410)					
Yes	15.8	56.6	10.9	16.7	<0.001*
No	16.5	47.9	10.7	24.9	

*p<0.05

Table S2. Bivariate analysis of weight status of children in the school year 2023–2024 sample (*N*=2347).

	**Underweight** %	**Healthy weight** %	**Overweight** %	**Obesity**	**p-value**
Household Food Insecurity (HFI) Risk (*n*=2323)					
Food Insecure	18.1	40.1	10.9	31.0	<0.001*
Food Secure	16.4	53.9	10.0	19.7	
Housing Status (*n*=2275)					
Rent	17.1	45.8	11.1	25.9	<0.001*
Own	16.5	55.6	9.5	18.3	
Single Parent (*n*=2240)					
Yes	17.5	43.8	9.6	29.1	<0.001*
No	16.9	53.6	10.3	19.2	
Household Income (*n*=2005)					
Less than $34,999	15.3	41.0	8.9	34.9	<0.001*
$35,000-$74,999	16.5	45.2	10.5	27.8	
More than $75,000	17.0	58.1	9.9	15.1	
Child Race (*n*=2347)					
White	18.8	56.1	9.5	15.6	<0.001*
Non-White	15.1	47.8	10.8	26.4	
Child Insurance (*n*=2335)					
Insured	17.1	51.4	10.4	21.2	0.088
Uninsured	11.5	50.4	8.0	30.1	
Primary Care Provider (*n*=2319)					
Yes	16.7	51.9	10.2	21.2	0.631
No	18.0	48.0	9.5	24.5	
Routine Check Up (*n*=2317)					
Yes	16.6	52.0	10.3	21.0	0.278
No	18.5	45.5	10.4	25.6	
Any Barriers Accessing Health Care (*n*=2307)					
Yes	13.5	51.6	9.5	25.3	0.064
No	17.4	51.7	10.4	20.5	
Physical Activity (Meeting Recommendations) (*n*=2294)					
Yes	16.0	55.5	9.4	19.1	0.003*
No	17.0	48.1	10.7	24.2	
Exclusive Breastfeeding at 6 months (*n*=2194)					
Yes	18.4	54.8	10.0	16.9	0.015*
No	16.5	50.5	10.2	22.9	
Breastfeeding at 12 months (*n*=2255)					
Yes	17.7	56.0	8.5	17.7	<0.001*
No	16.1	49.0	11.5	23.4	

*p<0.05

Table S3. Bivariate analysis of weight status of children in the school year (2024–2025) sample (*N*=2410).

	Underweight %	Healthy weight %	Overweight %	Obesity %	p-value
Household Food Insecurity (HFI) Risk (*n*=2388)					
Food Insecure	18.3	41.4	10.8	29.6	<0.001*
Food Secure	14.6	55.1	10.0	20.4	
Housing Status (*n*=2338)					
Rent	14.0	46.7	9.7	29.6	<0.001*
Own	16.1	57.5	10.4	16.1	
Single Parent (*n*=2315)					
Yes	14.1	40.7	13.8	31.4	<0.001*
No	15.6	56.4	9.2	18.9	
Household Income (*n*=2055)					
Less than $34,999	15.3	42.2	11.0	31.5	<0.001*
$35,000-$74,999	15.1	51.4	8.8	24.7	
More than $75,000	14.6	57.9	10.3	17.3	
Child Race (*n*=2410)					
White	15.3	58.6	10.8	15.3	<0.001*
Non-White	15.0	48.7	9.6	26.7	
Child Insurance (*n*=2394)					
Insured	15.0	53.1	10.1	21.8	0.714
Uninsured	19.1	48.9	10.6	21.3	
Primary Care Provider (*n*=2387)					
Yes	15.2	53.5	9.9	21.4	0.011*
No	15.0	41.2	13.1	30.7	
Routine Check Up (*n*=2378)					
Yes	14.5	54.0	10.0	21.5	<0.001*
No	21.9	38.1	11.3	28.7	
Any Barriers Accessing Health Care (*n*=2369)					
Yes	14.3	50.1	9.2	26.4	0.107
No	15.2	53.3	10.4	21.1	
Physical Activity (Meeting Recommendations) (*n*=2356)					
Yes	14.9	56.0	10.5	18.6	0.009*
No	15.4	50.9	9.5	24.2	
Exclusive Breastfeeding at 6 months (*n*=2257)					
Yes	16.7	56.0	9.8	17.5	0.005*
No	14.0	51.7	10.7	23.6	
Breastfeeding at 12 months (*n*=2304)					
Yes	16.2	55.3	8.9	19.6	0.035*
No	13.8	52.1	11.1	23.0	

*p<0.05

Table S4. Comparison of sociodemographic characteristics between excluded and included participants in the pooled sample (2022–2025).

	Included (*n*=7267)	Excluded (*n*=9316)	Test Statistic (df)	p-value
Household Food Insecurity (HFI) Risk				
Food Insecure	16.4%	25.7%	χ^2 (1) = 205.19	<0.001
Food Secure	83.6%	74.3%		
Housing Status				
Rent	41.8%	55.5%	χ^2 (1) = 293.94	<0.001
Own	58.2%	44.5%		
Single Parent				
Yes	22.4%	31.5%	χ^2 (1) = 162.41	<0.001
No	77.6%	68.5%		
Household Income				
Less than $34,999	16.0%	30.5%	χ^2 (2) = 607.86	<0.001
$35,000-$74,999	28.9%	34.1%		
More than $75,000	55.1%	35.5%		
Child Race				
White	44.1%	26.5%	χ^2 (1) = 560.76	<0.001
Non-White	55.9%	73.5%		
Uninsured	4.2%	7.4%		
